# Size- and Shape-Dependent Antibacterial Studies of Silver Nanoparticles Synthesized by Wet Chemical Routes

**DOI:** 10.3390/nano6040074

**Published:** 2016-04-15

**Authors:** Muhammad Akram Raza, Zakia Kanwal, Anum Rauf, Anjum Nasim Sabri, Saira Riaz, Shahzad Naseem

**Affiliations:** 1 Centre of Excellence in Solid State Physics, University of the Punjab, QAC, Lahore 54590, Pakistan; akaramraza.cssp@pu.edu.pk (A.R.); saira_cssp@yahoo.com (S.R.); shahzad.cssp@pu.edu.pk (S.N.); 2 Department of Zoology, Lahore College for Women University, Jail Road, Lahore 54000, Pakistan; zakia.kanwal@lcwu.edu.pk; 3 Department of MicroBiology & Molecular Genetics, University of the Punjab, QAC, Lahore 54590, Pakistan; anjum.mmg@pu.edu.pk

**Keywords:** AgNPs, reduction method, antibacterial activity, *Pseudomonas aeruginosa* (*P. aeruginosa*), *Escherichia coli (E. coli)*

## Abstract

Silver nanoparticles (AgNPs) of different shapes and sizes were prepared by solution-based chemical reduction routes. Silver nitrate was used as a precursor, tri-sodium citrate (TSC) and sodium borohydride as reducing agents, while polyvinylpyrrolidone (PVP) was used as a stabilizing agent. The morphology, size, and structural properties of obtained nanoparticles were characterized by scanning electron microscopy (SEM), UV-visible spectroscopy (UV-VIS), and X-ray diffraction (XRD) techniques. Spherical AgNPs, as depicted by SEM, were found to have diameters in the range of 15 to 90 nm while lengths of the edges of the triangular particles were about 150 nm. The characteristic surface plasmon resonance (SPR) peaks of different spherical silver colloids occurring in the wavelength range of 397 to 504 nm, whereas triangular particles showed two peaks, first at 392 nm and second at 789 nm as measured by UV-VIS. The XRD spectra of the prepared samples indicated the face-centered cubic crystalline structure of metallic AgNPs. The *in vitro* antibacterial properties of all synthesized AgNPs against two types of Gram-negative bacteria, *Pseudomonas aeruginosa* and *Escherichia coli* were examined by Kirby–Bauer disk diffusion susceptibility method. It was noticed that the smallest-sized spherical AgNPs demonstrated a better antibacterial activity against both bacterial strains as compared to the triangular and larger spherical shaped AgNPs.

## 1. Introduction

Nanoparticles are of tremendous importance in the field of nanotechnology by serving as basic building blocks in nanodevices for various practical applications. By virtue of a large surface-to-volume ratio and quantum confinement effect, nanoparticles exhibit unique and dramatically different physical, chemical, and biological properties relative to bulk materials [1]. Remarkable attraction and utilization of metallic nanoparticles in modern technologies results from their interesting surface plasmon characteristics, exciting physicochemical properties, and fascinating unique morphologies [2]. Metallic nanoparticles, therefore, are successfully playing their vital role in a variety of fields, such as information storage devices, photography, biological labeling, catalysis, photonics, optoelectronics, construction of magnetic ferrofluids, and surface enhanced Raman scattering [3]. Furthermore, they can also be used as a model system to experimentally investigate the effects of quantum confinement on magnetic, electronic and other related properties [4]. The fundamental characteristics of metallic nanoparticles strongly depend on their shapes, sizes, configurations, crystallinity, and structure whether they are in solid form or in hollow geometries [5]. Thus, by controlling such parameters one can achieve the desired properties of the nanoparticles.

Since ancient times silver has been used extensively for many applications such as jewelry, metalcraft, vessels or containers for liquid, coins, shavings, foils, and photography (where photosensitive Ag halides are reduced). Additionally, silver has been part of many medical applications throughout known history [6]. In the modern era, nano-sized silver particles are also a vibrant part of nanotechnology because of growing demands in various field; for example, chemical and biological sensing due to surface-enhanced Raman scattering properties, electronics and optoelectronics applications owing to the highest electrical and thermal conductivity among all other metals, energy harvesting, catalysis, imaging, and biomedicine [7,8].

One of the most important properties of AgNPS is their antimicrobial action against several bacteria, fungi, and viruses [9,10] and, thus, they are widely used as antimicrobial agents in different products, including clothes, plasters, bandages, toothbrushes, catheters, scalpels, cosmetics, needles, refrigerators, and mobile phones [11]. In fact, when AgNPs interact with microorganisms (bacteria, fungi, and viruses), silver ions (Ag^+^) are released and these ions may affect and damage the microorganism in different ways; for example, they attack the negatively-charged cell walls of the microbes to deactivate cellular enzymes and disrupt membrane permeability; consequently, cell lysis and cell death occurs [12,13]. Furthermore, the broad spectrum killing, oligodynamic action and lesser possibility for development of microbial resistance against AgNPs make them advantageous antibacterial agents. Surprisingly, AgNPs are safe and non-toxic to human and animal cells at low concentrations because the possible toxicity of AgNPs to the environment is considered extremely low as compared to other materials [14,15]. Perhaps, that is why AgNPs achieved the highest level of commercialization and account for 55.4% of the total nanomaterial-based consumer products available in the market (313 out of 565 products) [16,17]. Interestingly, the antimicrobial activity of nano-sized silver particles was found size- and shape-dependent, one of the reasons could be that different morphologies provide different areas to interact with microbes and thus results in different antibacterial efficiency [15,18,19].

AgNPs are prepared by various physical, chemical, and biological methods [8]. The most significant physical methods include arc-discharge, physical vapor condensation, and laser ablation [20,21]. The chemical methods frequently used for synthesis of nano-sized silver particles include chemical reduction, microemulsion, photoinduced reduction, UV-initiated photoreduction, photoinduced reduction, electrochemical synthetic approach, microwave-assisted synthesis, and irradiation methods [2,22,23]. The bio-based methods mostly consist of green synthesis approaches, where extracts of different plants, prokaryotic bacterial cells, and/or eukaryotic fungi are used as reducing agents to reduce the metallic silver precursor for the preparation of AgNPs [24].

In aforementioned techniques, physical methods normally demand sophisticated equipment so they may not be cost effective. For biological methods, in spite of their environment-friendly advantages, many critical aspects need to be considered; for instance, the nature of organisms, inheritable and genetic characteristics of organisms, suitable circumstances for cell growth and enzyme activity, optimum reaction environments, and choice of the biocatalyst to obtain the desired stable nanoparticles [2]. However, chemical methods, especially wet-chemical reduction techniques, can be considered the best approach owing to cost-effectiveness, simpler handling, low impurity factors, thermal stability, availability of chemicals for wide range nanoparticles synthesis, defined control over growth rate, and convenience to use different stabilizer to enhance the stability of prepared particles [1].

Although the antibacterial properties of silver nanomaterials are well accepted and documented, however, the debate on the topic that how the size and shape of nano-sized entities influence the antimicrobial performance is still ongoing. Since some of the researchers reported that anisotropic shapes of silver particles, such as nanoplates or triangular nanoprisms, played a key role to achieve high biocidal activity. For example, Pal, *et al.* [15] showed that truncated triangular AgNPs exhibited better antibacterial efficiency than that of the spherical and rod-shaped silver particles. Dong, *et al.* [19] presented their results claiming that sharp edge and sharp vertex triangular silver nanoprisms showed the best antiseptic performance as compared to spherical and near spherical particles. Similarly, Sadeghi, *et al.* [25] also described that the silver nanoplates demonstrated the higher antibacterial effectiveness than silver nanopsheres or nanorods. One of the reasons given for such great antibacterial activity of these anisotropic-shaped AgNPs was the basal plane with high-atom-density {111} facets which acted as the maximum reactivity sites leading to the strongest antibacterial activity [26].

On the other hand, some reports [2,27,28] indicated that isotropic geometries such as spherical particles also demonstrated high antibacterial effectiveness. Their main argument was large surface to volume ratio of spherical shapes, which provided the maximum reactivity to obtain the highest antibacterial activity. Therefore, the investigations to understand the influence of nanoparticles with different geometry size, chemical functionality, and surface charge on biological systems is of great importance [29].

The aim of the present study was to explore the effect of AgNPs having different shapes and sizes against two Gram-negative bacteria. We synthesized the triangle- and sphere-shaped AgNPs by wet chemical routes and characterized them by standard characterizing techniques as UV-VIS, XRD, and SEM. The antibacterial properties of produced AgNPs were studied *against Pseudomonas aeruginosa* (*P. aeruginosa*) and *Escherichia coli* (*E. coli*) by disk diffusion methods. The antibacterial effects of prepared silver particles were determined by zone of inhibition (ZOI) for both bacterial strains. The obtained results were analyzed and discussed in light of available literature.

## 2. Results

### 2.1. Silver Nanoparticles Production

**Figure 1:**
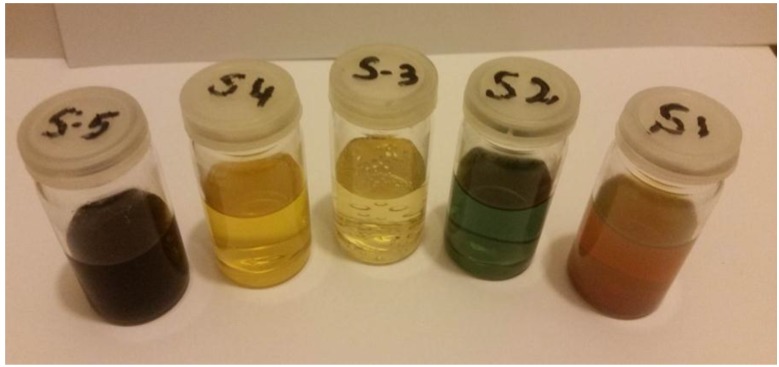
The manifestation of different colors of silver nanoparticle samples, the type of sample is written on the lid of each sample bottle.

Figure 1 shows the beautiful colors of prepared five different AgNPs samples labeled as S1, S2, S3, S4, and S5; different colors depict the different characteristics of silver nanoentities. The preparation summary for each sample is listed in Table 1.

The mechanism reduction method to prepared different types AgNPs can briefly be described as silver nitrate (precursor) was dissolved into the water, silver ions (Ag^+^) were produced. The addition of reducing agents, such as sodium citrate and/or sodium borohydride, created free metallic silver (Ag^0^) atoms by the reduction of silver ions. As the reaction proceeded, under the influence of reaction conditions, such as constant stirring and temperature, these silver atoms (Ag^0^) accumulated into the oligomeric clusters and finally these clusters led to the foundation of silver colloids [30]. The role of the PVP (polyvinylpyrrolidone, a stabilizing agent) was to stabilize the formed AgNPs and to prevent them from absorbing or attaching with each other’s surfaces and, thus, to avoid the agglomeration of nanoparticles during synthesis. The hydrogen peroxide provided the required assistance to induce further oxidation of small particles into Ag^+^ for the formation of different sizes and shapes [19,28].

### 2.2. UV-VIS Spectroscopy Investigations

**Figure 2:**
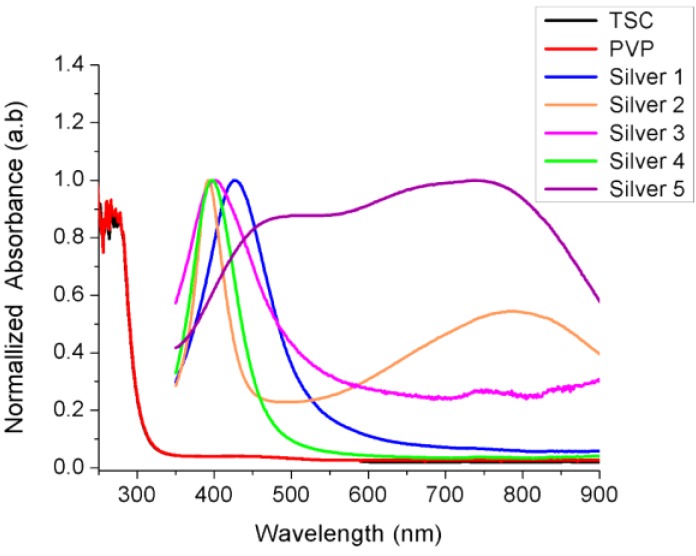
Ultraviolet visible (UV-VIS) absorption spectra of all samples showing different surface plasmon resonance (SPR) peaks.

The optical and structural properties of all samples were determined by UV-VIS and obtained absorption spectra are shown in Figure 2. The absorption spectra of tri-sodium citrate (TSC) and PVP showed no absorption peak in entire visible region which is according to the already reported results [31,32]. Sample 1 (S1, greenish yellow) showed a single surface plasmon resonance peak at the value of 426 nm, the full width at half maximum (FWHM) value of this peak measured as 107 nm. This indicated that particles were spherical with a somewhat wide range of size distribution.

The UV-VIS spectra of the dark green solution (sample S2) displayed two characteristic peaks at 392 nm and 789 nm, respectively. The first peak was very sharp with FWHM of only 53 nm while the second peak was broader one. The characteristic absorption peaks peak for Sample 3 (light yellow silver colloid) occurred at 403 nm with a larger FWHM value of 152 nm. In the case of the bright yellow AgNPs solution (S4) again a sharp single SPR peak appeared at 397 nm with a FWM value of 73 nm. However, Sample 5 (dark blue solution) showed very broad peak with three shoulders appeared at 504 nm, 678 nm, and 735 nm and value of FWHM was about 544 nm. A summary of all UV-VIS results is listed in Table 2.

### 2.3. Scanning Electron Micrscopy Analysis

**Figure 3:**
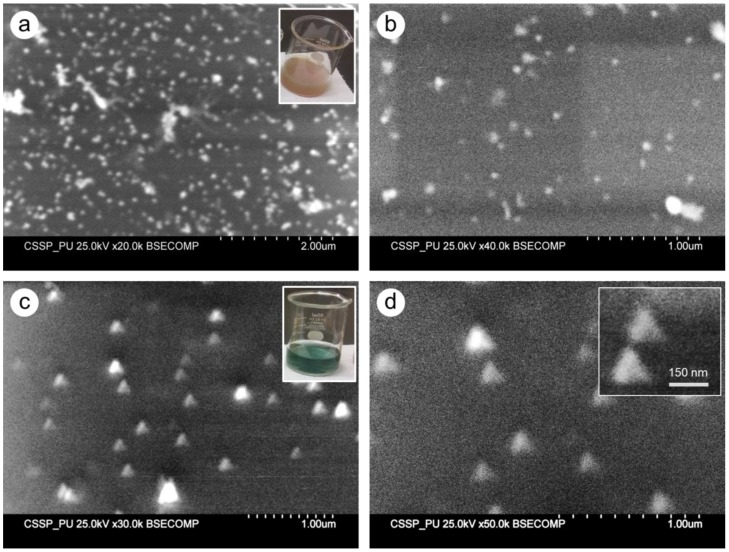
SEM images of Sample 1 at lower (**a**) and higher (**b**) magnification showing the spherical morphology of the particles. The triangle-shaped silver particles are represented in (**c**) and (**d**) at different magnifications. The inset showed the color of sample S1 and sample S2 solution the beaker.

The shape and size of the prepared samples were determined by SEM. In Figure 3, the shape and size distribution of sample S1 and S2 are displayed. The deposition of AgNPs on glass substrate for sample S1 is shown in Figure 3a,b at different magnification. At some places the few clusters (Figure 3a) of AgNPs indicated the agglomeration of nanoparticles during the deposition procedure. Figure 3b shows the magnified view and the spherical morphology is evident. The size of S1 AgNPs was found in the range of 30 nm to 80 nm. The morphological study of sample S2 is illustrated in Figure 3c,d, which revealed the interesting distribution of triangle shaped silver particles. A few of the triangular particles appeared larger, while most of the triangles were of same size. The average edge-length of these particles was found 150 nm. In a magnified view (Figure 3d) it can be seen that most of the triangular particles had nice, sharp edges and vertexes, while a few appeared as truncated nano-triangles. The inset in Figure 3d displayed two typical silver nano-triangles; the scale bar provides the idea of the size of triangular particles.

**Figure 4:**
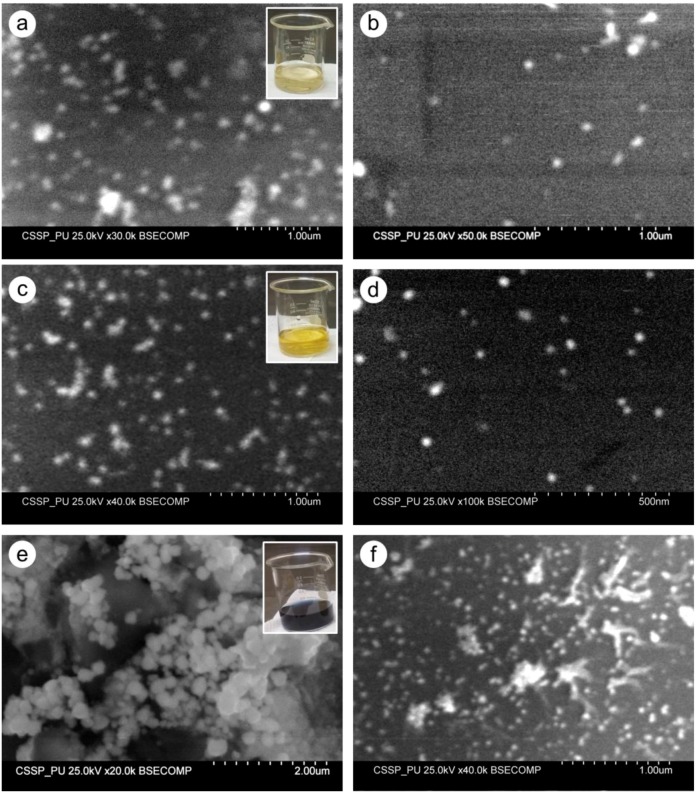
SEM micrographs of sample S3 (**a**,**b**), S4 (**c**,**d**), and S5 (**e**,**f**) presenting the shape and size of preapred AgNPs. The inset showed the the color of correspoding colloidal sample.

The SEM photographs of sample S3 are shown in Figure 4a,b at different magnifications. Most of the particles appeared spherical with a broad size distribution from 25 to 70 nm. At some places on the substrate the aggregation of the particles can be seen (Figure 4a). In the case of sample 4 (S4) spherical particles were found much smaller with size distribution ranging from 15 nm to 50 nm as shown in Figure 4c,d. However, few larger spherical and near-spherical objects were also present, which could be the result of agglomeration of particles at some places on the glass substrate. The SEM photographs of sample S5 showed aggregation of silver particles into clusters and bunches. The morphology of these bigger objects appeared spherical and irregular multi-branched particles (Figure 4e). The size measurement showed that these particles were in range of 30 nm to more than 200 nm.

### 2.4. X-Ray Diffraction Pattern

**Figure 5:**
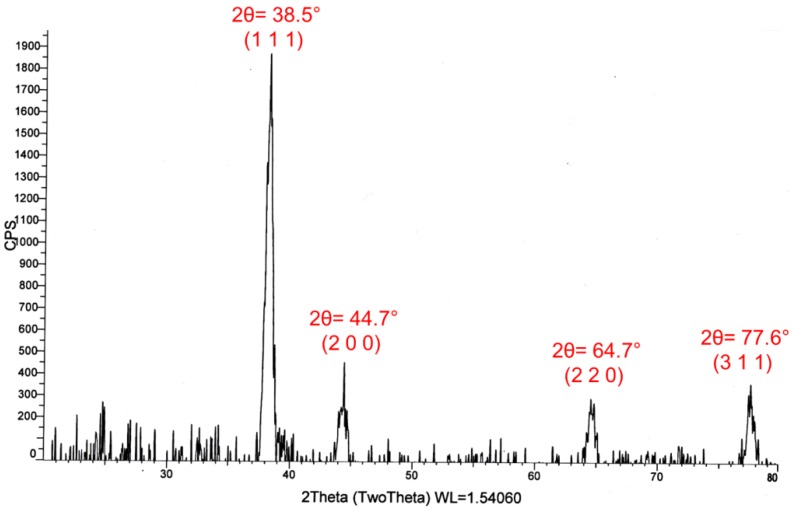
Xray difraction (XRD) pattern of sample 1 (S1), showing the face centered cubic (FCC) crystalline metallic silver nanoparticles (AgNPs). The intesity in vertical axis is mearred in counts per second (CPS) and diffraction angle (2 theta) measred is taken along horizental axis. The value of wavelngth (WL in angstrom) is also mentioned in the figure.

In order to obtain the crystalline information of synthesized AgNPs, X-ray powder diffraction analysis was carried out. The XRD pattern of sample (S1) is shown in Figure 5. Four sharp peaks appeared at 2θ = 38.5°, 44.7°, 64.7°, and 77.6°, which can be assigned to the (111), (200), (220), and (311) planes of the face centered cubic (FCC) structure of metallic silver, respectively, according to the JCPDS File No. 04-0783 [33]. The average crystal size as calculated by the Debye–Scherrer formula [34] was found *d* = 40 nm. Furthermore, the most intensive peak located at 2θ = 38.5° corresponding to the diffractions of spherical nanoparticles crystallized in the FCC structure with basal {111} lattice plane.

### 2.5. Antibacterial Activity Study

**Figure 6:**
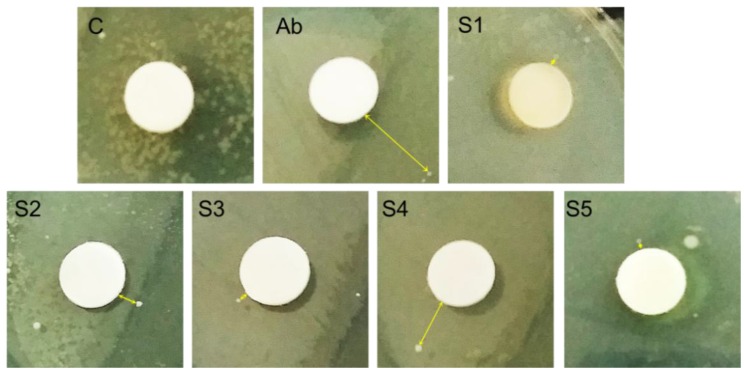
*Pseudomonas aeruginosa* zones of inhibition (ZOI) around silver nanoparticles (AgNPs) impregnated disks. The distance of the first colony from the disk/ZOI is demonstrated by arrow headed lines.

**Figure 7:**
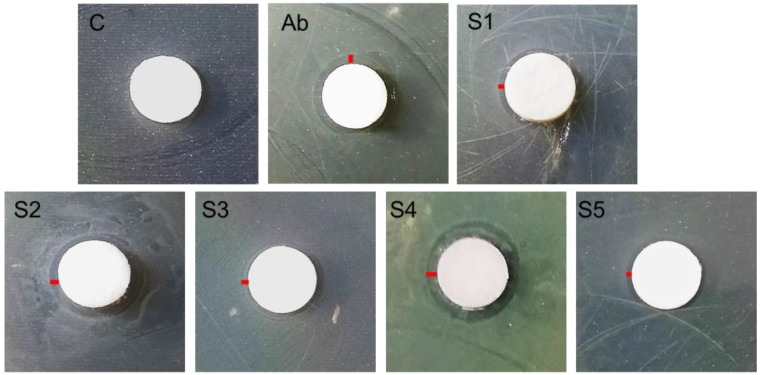
*Escherichia coli* zones of inhibition (ZOI) around silver nanoparticles (AgNPs) impregnated disks. The distance of the bacterial lawn from disk/ZOI is demonstrated by red lines.

To evaluate the shape and size dependent bactericidal action of produced AgNPs on *P. aeruginosa* and *E. coli*, the disk diffusion method was followed. Sterile paper disks impregnated in different AgNPs were placed on the nutrient agar plates on which bacteria were spread. After 24 h of incubating the plates at 37 °C, the resulting growth of bacteria was detected. The obtained results for bacterial growth on the agar plates in the presence of AgNPs impregnated disks are illustrated in Figure 6 for *P. aeruginosa* and in Figure 7 for *E. coli*. Probably the phase of the growth, in the case of *P. aeruginosa* in the first 24 h of incubation, was in the late log phase as reported by Wu, *et al.* [35]. In the case of *E. coli*, the growth phase in the first 24 h of incubation could be a stationary phase [36,37]. In the case of *P. aeruginosa* the distance (mm) of the first colony formed from the disk was noted and taken as Zone of Inhibition (ZOI). It can be seen from Figure 6 that in case of negative control (C) which was pure water impregnated disk, there was a huge growth of bacterial colonies all around the disk and no distance (0 mm) between disk and the colonies can be seen. In case of antibiotic Ciprofloxcin (Ab) the distance between the Ab disk and the first bacterial colony was 11.3 mm. The ZOI for all the AgNPs, S1, S2, S3, S4, and S5 were found to be 1 mm, 3 mm, 1.6 mm, 8 mm, and 0.8 mm, respectively. Thus, the maximum antibacterial activity shown in our assay was that of antibiotic (11.3 mm) and second highest efficiency was of sample S4 (8 mm), however all types of AgNPs showed antibacterial action against *P. aeruginosa*. All the measurements are listed in Table 3. It is evident from Figure 6 and Table 3 that sample S4 (smaller spherical AgNPs) demonstrated the strongest bactericidal activity while sample S5 (larger spherical and near spherical AgNPs) showed the minimum activity. Interestingly, antibacterial performance of triangular AgNPs (S2) against *P. aeruginosa* was found to be lesser than that of smallest spherical AgNPs (S4).

**Figure 8:**
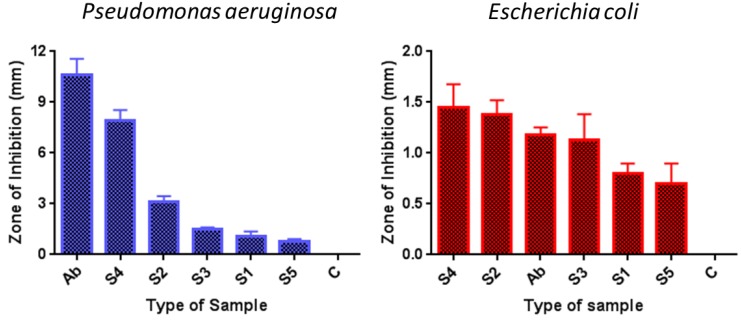
Antibacterial activity (high to low) of silver nanoparticles (AgNPs) against *Pseudomonas aeruginosa* and *Escherichia coli.*

In the case of *E. coli*, clear circular Zones of Inhibition (ZOI) were formed around the disks (Figure 7). The diameter of ZOI (mm) was noted for each disk and listed in Table 3. No ZOI was formed around the disk impregnated in water (C). In case of antibiotic Ciprofloxacin (Figure 7) the ZOI was 1.2 mm. The measured ZOI for AgNPs samples S1, S2, S3, S4, and S5 were 0.9 mm, 1.4 mm, 1.1 mm, 1.5 mm, and 0.7 mm, respectively. Furthermore, in the case of *E. coli*, the maximum antibacterial activity was shown by sample S4 (1.5 mm) which was even greater than the antibiotic Ciprofloxacin (1.2 mm). Interestingly, again the antibacterial activity of triangle shaped silver particles (S2) was lower (1.4 mm) than that of smaller spherical AgNPs (1.5 mm) but more than the antibiotic (1.2 mm). Thus, a similar trend was observed that sample S4 displayed the strongest, while sample S5 demonstrated the lowest, bacteria-killing activity. Nevertheless, all AgNPs samples showed antiseptic action against *E. coli* as listed in Table 3. A series (from highest to lowest) of antibacterial effectiveness of all samples against both bacterial strains is shown in Figure 8.

## 3. Discussion

### 3.1. Synthesis and Characterization Analysis

During the synthesis of different AgNPs, the metallic precursor (silver nitrate, in this case) provided Ag^+^ ions which were reduced to free silver atoms (Ag^0^) by gaining the electrons under effect of reducing agents (tri-sodium citrate or sodium borohydride). Due to nucleation and growth processes, these free silver atoms accumulated to form AgNPs [28]. The role of stabilizing agent (PVP) was very important in the stabilization of silver ions which consequently lead to control the size and shape of particles. Under the combined influence of different reducing, stabilizing, and/or oxidizing agents, the shape and size of AgNPs developed which, subsequently, resulted in different colors of the solution as shown in Figure 1. Furthermore, the reaction conditions may also play the crucial role in achieving different morphology and/or size of particles. As we observed in the preparation of sample S2 and sample S5, all the reagents were the same except the stirring timings. Perhaps the continuous stirring (in case of S2) facilitated metallic silver to attain the desired sites for developing triangular-shaped particles (Figure 3c). While in the case of sample S5, absence of stirring caused silver atoms to agglomerate into larger silver clusters as shown in Figure 4e. Our arguments are also supported by the study of Li, *et al.* [38]. They found that diverse stirring conditions resulted in different sizes and morphology of nanoparticles due to different mechanical agitation [38]. We suggest that further investigations may provide more information to figure out the true role of stirring in this regard.

Different colors of colloidal silver samples indicated different size and morphology of synthesized nanoparticles. This was further characterized by UV-VIS and SEM techniques. The single SPR peaks of sample S1, S3, and S4 (Figure 2) at 426 nm, 403 nm, and 397 nm, respectively, depicted the spherical AgNPs with sizes S1 > S3 > S4. This was confirmed by SEM analysis of the samples that all three samples were almost spherical morphology with size distribution slightly different as suggested by UV-VIS spectra. Apparently the size measured from the SEM images seems larger than predicted by UV-VIS spectra. For example in the case of sample S1 and sample S4, the characteristics peaks at 426 nm and 397 nm predicted the size of AgNPs 45 nm and 10 nm, respectively, as reported by Bastus, *et al.* [7]. In our case the larger size of AgNPs appeared in SEM photographs could be due to the possible agglomeration of the particles on the glass substrate during the deposition process.

In case of sample S2 (dark green solution), instead of a single peak, two characteristics peaks appeared at 397 nm and 789 nm indicating the anisotropic morphology of the AgNPs. These peaks suggested the induced polarizations; peak at 397 nm indicated the out-of-plane dipole resonance while peak at 789 nm designated in-plane dipole plasmon resonance. These peaks suggested the triangular shaped structure formation. Moreover the peak at around 789 nm indicated the perfect sharpness of vertexes of our silver triangle shaped particles [39]. The SEM results confirmed the UV-VIS interpretation for sample S2 revealing the triangular silver particles with sharp tips (Figure 3c,d). The sample S5, showed a very wide peak with contours appearing at 504 nm, 678 nm, and 735 nm. The lack of sharpness in the peaks suggested the irregular larger silver objects as was seen in SEM images (Figure 4e).

The XRD results of sample 1 showed the lattice parameter 4.06 Å which is close to the literature value 4.086 Å [30]. The crystallite size of S1 was found 40 nm and SEM results also confirmed that average size measured was 45 nm.

### 3.2. Effect of Shape and Size on Antibacterial Activity

We evaluated the antibacterial performance of AgNPs samples against *P. aeruginosa* and *E. coli*. It can be seen from the antibacterial activity analysis that all AgNPs demonstrated the bactericidal function against both bacterial strains (Figure 6 and Figure 7). However, it is evident as illustrated in Figure 8 that the antibacterial efficiency of all silver samples against *E. coli* was low as compared to *P. aeruginosa*. This indicated that the *P. aeruginosa* was more susceptible than *E. coli*. Sample S4, the smallest-sized spherical AgNPs, exhibited the maximum bactericidal efficacy against both bacterial strains while sample S2, the triangular AgNPs, showed the second highest antibacterial activity in both bacterial studies. Furthermore, antibacterial performance of samples S4 and S2 were observed to be even better than Ciprofloxacin (Ab), which suggests that silver nanoparticles can be a good alternative for antibiotics which have resulted in greater bacterial resistance. Interestingly, the overall antibacterial efficiency trend of all five samples against *P. aeruginosa* and *E. coli* was found similar and can be listed as S4 > S2 > S3 > S1 > S5 (Table 3, Figure 8). This indicated that the smallest-sized spherical AgNPs (S4) were more efficient to kill and destroy both types of bacteria as compared to larger spherical AgNPs (S3, S1, and S5). When paper disks were impregnated with colloidal silver particles of different size and shape, the rate of dissolution of silver cations for various particles was different. Due to the high surface to volume ratio, the smaller-sized nanoparticles released more silver cations and, thus, proved more effective to kill the bacteria as compared to larger-sized particles. These results are in accordance with already reported outcomes [2,27].

AgNPs may interact with microorganisms in many ways to damage them. For example, AgNPs may release silver ions when come in contact with bacterial cells. These ions may affect the bacterial DNA replication functions; deactivating the production of some enzymes and cellular proteins necessary for adenosine tri-phosphate (ATP) synthesis [2,15]. Furthermore, silver ions may disrupt the respiratory chain by disturbing the working of membrane-bound enzymes [40]. The smaller spherical AgNPs showed better inhibitory action because a significantly large surface area was in contact with the bacterial effluent owing to the larger surface to volume ratio as compared to larger spherical AgNPs. Thus, smaller particles released more silver ions than larger particles to kill more bacteria [15,41].

Surprisingly, the triangle shaped silver particles (S2) demonstrated less antibacterial activity than that of the smaller spherical particles (S4). Apparently, our results seem different than those reported by Pal, *et al.* [15] and Dong, *et al.* [19] who described the strongest antibacterial activity of triangular shaped silver nonparties as compared to spherical ones. They argued that high reactivity of triangular AgNPs was due to their geometrical structure and {111} crystal planes. These high-atomic-density {111} facets lead to maximum antibacterial productivity.

The higher biocidal efficacy of our smaller spherical AgNPs (S4) as compared to our triangular AgNPs (S2) can be explained in various ways. For example, in the XRD pattern (Figure 5) of spherical AgNPs, the most intense diffraction peak appeared at 2θ = 38.5° from the {111} lattice plane indicated that spherical AgNPs had the top basal plane with {111} facets. This suggested that the smaller spherical AgNPs (S4) might also have the high-atomic-density {111} facets which acted as active sites. Thus, large surface to volume ratio and high-atomic-density {111} facets perhaps enhanced the bacterial killing efficiency of S4 as compared to S2. Moreover, the smaller-sized spherical AgNPs (S4) were more effective to penetrate inside the bacteria as compared to the larger triangular-shaped AgNPs [42]. Inside the bacteria, the spherical AgNPs, being a soft acid, probably interacted and destroyed the sulfur- and phosphorus-containing complexes (soft bases) like DNA, and also disrupted the morphology of the membrane, finally leading to the cell death [2,15,26,40].

Although our results demonstrated that smallest sized spherical AgNPs were the best antibacterial agents among triangular and larger spherical AgNPs, nevertheless, we suggest more investigations to fully explore the shape- and size-dependent biocidal activity of AgNPs because the role of effective surface areas of different geometries is still not fully understood [15]. We hope that our study on size- and shape-dependent bactericidal efficacy could facilitate a new paradigm for considering the true role of AgNPs as antimicrobial agents in drug formulation.

## 4. Materials and Methods

### 4.1. Materials

Silver Nitrate (AgNO_3_, Molecular weight (Mw): 169.87 g/mol), tri-sodium citrate (Na_3_C_6_H_5_O_7_, Mw: 294.10 g/mol), sodium borohydride (NaBH_4_, Mw: 37.83 g/mol), hydrogen peroxide (H_2_O_2_ 30%), and polyvinylpyrrolidone (PVP; Mw: 1,300,000) were of analytical grade from Merck (Darmstadt, Germany). Ciprofloxacin (Bayer-Leverkusen, Germany) was purchased from a local medical store. Highly-purified deionized water was used throughout the experiment. The bacterial strains used for antibacterial activity was obtained from Department of Microbiology and Molecular Genetics (MMG), University of the Punjab, Lahore, Pakistan.

### 4.2. Preparation of Silver Nanoparticles

Five different samples of AgNPs were synthesized by wet chemical reduction methods following the procedure of Dong, *et al.* [19] with some modifications. A summary of experimental details including reagents with quantities are listed in Table 1.

#### 4.2.1. Sample 1 (S1)

**Figure 9:**
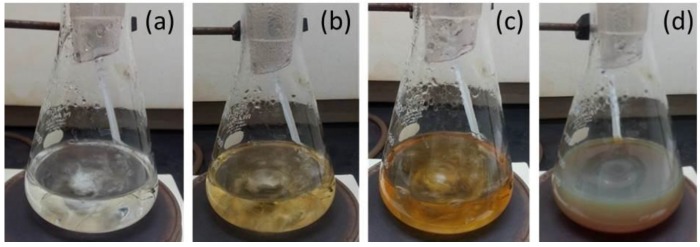
Color changing of sample 1 at different phases of the reaction. (**a**) Transparent color appeared on dissolving silver nitrate into the water to form silver ions at boiling temperature under continuous stirring; (**b**) light yellow color indicated the reduction of silver ions into very small silver particles after the addition tri-sodium citrate; (**c**) bright yellow color depicted the formation of larger silver particles from the smaller ones; (**d**) finally greenish yellow color revealed the completion of the reaction when all silver ions had been reduced into the elemental silver nanoparticles by the tri-sodium citrate.

50 mL of 1 mM solution of AgNO_3_ prepared in water was heated to the boiling temperature under vigorous stirring to dissolve completely. With the help of dropper, 5 mL of 1% tri-sodium citrate (Na_3_C_6_H_5_O_7_) aqueous solution was added dropwise into the boiling AgNO_3_ solution. The reaction was completed at boiling point under constant stirring and refluxing condition. The color of the solution changed at different stages during the reaction (as shown in Figure 9) from transparent (Figure 9a), to pale yellow (Figure 9b), to bright yellow (Figure 9c), and finally greenish yellow (Figure 9d) which indicated the completion of the reaction. The solution was allowed to cool at room temperature under stirring. Since the tri-sodium citrate after oxidation became the stabilizer these particles (S1) can also be known as citrate-stabilized silver nanoparticles.

#### 4.2.2. Sample 2 (S2)

First of all 1 mL of 5 mM AgNO_3_ solution was added in 50 mL deionized water during stirring (400 rpm), then 0.5 mL of 1 mM PVP (Mw: 1,300,000) was added in above solution at room temperature. After 10 min 3 mL of 30 mM tri-sodium citrate and 0.2 mL of hydrogen peroxide was added under constant stirring. After 30 s 0.5 mL of 50 mM NaBH_4_ was added. After about 30 min, the solution changed from faint yellow to dark green color. The reaction was continued for 5 h under constant stirring (400 rpm).

#### 4.2.3. Sample 3 (S3)

50 mL of 2 mM NaBH_4_ aqueous solution was prepared and ice cooled under constant stirring (400 rpm) for 30 min. 2 mL of 1 mM AgNO_3_ solution was then added dropwise with the help of dropper at the rate of one drop per second. Stirring was stopped as soon as all the AgNO_3_ was added in the solution. The whole reaction was carried out at room temp.

#### 4.2.4. Sample 4 (S4)

0.5 mL of 30 mM tri-sodium citrate was added into 50 mL deionized water under constant stirring (400 rpm) at room temp and allowed to dissolve it completely. Afterwards 1 mL of 5 mM AgNO_3_ was added to above solution. Before adding freshly prepared 0.5 mL of 50 mM NaBH_4_ aqueous solution quickly, the stirring was stopped. The color of the solution changed to light yellow. After 30 s, 0.5 mL of 1 mM PVP (Mw: 1,300,000) aqueous solution was added and reaction continued for another 30 min. The color turned into bright yellow at the completion of reaction.

#### 4.2.5. Sample 5 (S5)

All procedures and reagents were the same as in the case of sample 2 synthesis. Only reaction conditions were changed, the whole experiment was carried out in the dark, and at the stage when NaBH_4_ was added the stirring was stopped before adding NaBH_4_. After 30 min, the color of the solution changed from faint yellow to dark blue. The reaction was allowed to continue for 5 h.

### 4.3. Characterization of Prepared Silver Nanoparticle Samples

To determine various characteristic of formulated AgNPs, different techniques were used. To study the optical absorption properties of different silver colloids, ultraviolet–visible spectroscopy (Nicolet, Evolution 300, Thermo Electron Corporation, Waltham, MA, USA) was used at room temperature in air. We used the X-ray powder diffractometer (Model: D-maxIIA, Rigaku, Tokyo, Japan) to analyze the structural properties and crystallite size. For the XRD sample, a few droplets of a typical sample (S1) were dried on the glass substrate to form a thick film. The size and shape analysis of prepared AgNPs was carried out by scanning electron microscope (Model: S3400N, Hitachi, Tokyo, Japan). For the SEM samples, different approaches were practiced to deposit silver nanoparticles on the glass substrate, such as the drop casting method and substrate immersion method.

### 4.4. Antibacterial Activity Tests

The antibacterial susceptibility of prepared AgNPs against two Gram-negative bacterial strains; *P. aeruginosa* and *E. coli* was evaluated by disk diffusion/Kibry–Bauer method (17). Briefly, a 100 μL sample of freshly-grown bacterial suspension (with a concentration of ~10^4^ and ~10^6^ colony forming unit (CFU)/mL of *P. aeruginosa* and *E. coli*, respectively) cultured in LB (Luria Bertani) was spread on the nutrient agar plates. Small sterile paper disks of uniform size (10 mm) were impregnated with as prepared AgNPs colloidal samples and then placed on the nutrient agar plates. Disks impregnated with Ciprofloxacin and pure water were also placed on nutrient agar for positive (Ab) and negative (C) controls, respectively. Plates were then incubated at 37 °C for 24 h. The resulting bacterial colonies’ distance/inhibition zones around the disks were then recorded.

## 5. Conclusions

We successfully prepared triangular AgNPs and spherical nanoparticles of different sizes. The characterization of the prepared nanoparticles was carried out by SEM, UV-VIS. and XRD. UV-VIS provided the morphological and size information by absorption spectra. SEM images confirmed spherical and triangular shapes of AgNPs. XRD indicated the FCC crystalline structure of prepared AgNPs. The antibacterial inhibition tests showed that all of our AgNPs were toxic to both *P. aeruginosa* and *E. coli* and their antibacterial efficacy was found size and shape dependent. The smaller-sized spherical AgNPs demonstrated higher antiseptic efficacy than that of triangular AgNPs, whereas larger spherical AgNPs were found less efficient in bactericidal action than triangle shaped AgNPs against both bacterial strains. Two of our samples, S2 and S4, showed more bactericidal activity against *E. coli* than Ciprofloxacin, which suggests that AgNPs with optimized size and shape could be a potential alternative for antibiotics which have encountered more bacterial resistance.

## Figures and Tables

**Table 1:** Summary of experimental work for the preparation of silver nanoparticles (AgNPs). Silver nitrate (AgNO_3_), tri-sodium citrate (TSC, Na_3_C_6_H_5_O_7_), sodium borohydride (NaBH_4_), hydrogen peroxide (H_2_O_2_) and polyvinylpyrrolidone (PVP) were used for the synthesis of silver nanoparticles.

Sample	Precursor	Reducing Agent	Stabilizing Agent	Oxidizing Agent	Reaction Conditions
S1	AgNO_3_ (1 mM, 50 mL)	Na_3_C_6_H_5_O_7_ (1%, 5 mL)_,_ added dropwise	TSC	-	At boiling temp, vigorous continuous stirring, finally cooled at room temp
S2	AgNO_3_ (5 mM, 1 mL)	NaBH_4_ (50 mM, 0.5 mL), Na_3_C_6_H_5_O_7_ (30 mM, 3 mL)	PVP (0.5 mL, 1 mM), TSC	H_2_O_2_ (0.2 mL)	At room temp, continuous stirring (400 rpm) through the experiment
S3	AgNO_3_ (1 mM, 2 mL), Added dropwise	NaBH_4_ (2 mM, 50 mL)	-	-	At ice cooling, stirring (400 rpm) until all AgNO_3_ was added
S4	AgNO_3_ (5 mM, 1 mL)	NaBH_4_ (50 mM, 0.5 mL), Na_3_C_6_H_5_O_7_ (30 mM, 0.5 mL)	PVP (0.5 mL, 1 mM), TSC	-	At room temp, NaBH_4_ was added quickly, Stirring (400 rpm) was stopped before adding NaBH_4_
S5	AgNO_3_ (5 mM, 1 mL)	NaBH_4_ (50 mM, 0.5 mL), Na_3_C_6_H_5_O_7_ (30 mM, 3 mL)	PVP (0.5 mL, 1 mM), TSC	H_2_O_2_ (0.2 mL)	At room temp, in dark, stirring (400 rpm) was stopped before adding NaBH_4_

**Table 2:** Summary of ultraviolet visible (UV-VIS) spectroscopy and scanning electron microscopy (SEM) measurements. (FWHM: full width at half maximum)

Sample	Peaks	FWHM	Color	Shape	Size
S1	426 nm	107 nm	Greenish yellow	Spherical	30–80 nm
S2	392 nm	53 nm	Dark green	Triangular	Edge-length 150 nm
	789 nm				
S3	403 nm	152 nm	Light yellow	spherical	25–70 nm
S4	397 nm	73 nm	Bright yellow	spherical	15–50 nm
S5	504 nm	544 nm	Dark blue	spherical	30–200 nm
	678 nm				
	735 nm				

**Table 3:** Average zone of inhibition (mm) of silver nanoparticles (AgNPs) against *Pseudomonas aeruginosa* (*P. aeruginosa*) and *Escherichia coli* (*E. coli*).

Sample	*P. aeruginosa*	*E. coli*
C	0	0
Ab	11.3 ± 0.8	1.2 ± 0.1
S1	1 ± 0.2	0.9 ± 0.15
S2	3 ± 0.2	1.4 ± 0.2
S3	1.6 ± 0.1	1.1 ± 0.35
S4	8 ± 0.5	1.5 ± 0.3
S5	0.8 ± 0.1	0.7 ± 0.3

## Abbreviations

The following abbreviations are used in this manuscript:

AgNPs	Silver nanoparticles
TSC	Tri-sodium citrate
SEM	Scanning electron microscopy
XRD	X-ray Diffraction
ZOI	Zone of Inhibition
FCC	Face Centered Cubic
